# Classification of Electronic Waste Components through X-ray and Neutron-Based Imaging Techniques

**DOI:** 10.3390/ma17194707

**Published:** 2024-09-25

**Authors:** Noémi Anna Buczkó, Mariann Papp, Boglárka Maróti, Zoltán Kis, László Szentmiklósi

**Affiliations:** 1 Nuclear Analysis and Radiography Department, HUN-REN Centre for Energy Research, 1121 Budapest, Hungary; 2 Hevesy György PhD School of Chemistry, ELTE Eötvös Loránd University, 1117 Budapest, Hungary

**Keywords:** X-ray imaging, neutron imaging, image segmentation, non-destructive characterization

## Abstract

In modern society, the amount of e-waste is growing year by year. Waste electronic items are complex, highly heterogeneous systems, containing organic material as well as several exotic, valuable, toxic, mostly metallic elements. In this study, the potential of X-ray and neutron radiography to reveal the inner structure of various complex e-waste was investigated. The images obtained using the two techniques were evaluated together to investigate the possibility of a more efficient segmentation of the individual components. The advantages and limitations of the two methods were identified for the studied waste types. X-ray radiography was found to be preferable for the identification of small metallic parts and for revealing the internal structure of e-waste with thick plastic coatings. Neutron radiography allowed for the identification of several components that did not provide sufficient contrast with X-ray imaging due to their similar X-ray attenuation compared to their surroundings. The combination of the two methods opens up new opportunities and could provide much more effective segmentation than either method alone.

## 1. Introduction

Due to the increasing demand for consumer goods and their shortening life cycle, the amount of waste electrical and electronic equipment (WEEE, or e-waste) is rapidly growing [1,2,3]. The management and recycling of e-waste must address three aspects, often classified as toxic, valuable, and critical materials. The contained toxic substances have an impact on human health and the environment; therefore, regulations exist worldwide to limit the hazardous components of commercial products (e.g., Restriction of Hazardous Substances (RoHS) [4], Waste Electrical and Electronic Equipment Directive (WEEE) [5]). Secondly, during production, substances with high economic value (such as Au, Ag, Pd, and Pt) are used; therefore, the waste, if recycled, can be a valuable and profitable secondary source of these elements, in contrast to conventional mining activity. New electronic gadgets and appliances are notably reliant on exotic materials (e.g., rare earth elements in magnets [6], In and Ga in flat screen displays [6,7]), while there is a shortage in the worldwide supply chain [8], so recycling them can help to sustain the local availability of these elements (Critical Raw Materials (CRM) Initiative of the EU) [9]. The recognition of these principles has led to the present concept of a circular economy [10].

Technically, WEEE is a complex system, containing various organic components and metals. For instance, a typical PCB contains ~28% metals (10–12% Cu, 4–6% Pb/Sn, plus Ga, In, Ti, Si, Ge, As, Sb, Se, Te, Ta, Pt), ~23% plastics, and the remaining percentage as ceramics and glassy materials [11]. The processing of waste IT equipment usually starts with shredding, which is followed by separation based on physical properties (magnetic separation [12], electrical conductivity), thermal treatment (melting, pyrolysis [13,14], smelting [15], incineration [16]), or a hydrometallurgical process [14,17].

None of these techniques are particularly environment-friendly: thermal treatment and electrolysis are energy intensive, while the hydrometallurgical technology uses toxic and aggressive chemicals, e.g., mixtures of aqua regia, hydrogen peroxide, and hydrofluoric acid [14,18]. For specific types of waste, therefore, sorting and disassembling are a more sophisticated strategy than the above-listed methods, as the valuable, toxic, and critical elements are concentrated in certain components of the waste items.

Before disassembling the discarded items, a preparatory separation step is conducted manually, or via automated sorting devices integrated with a conveyor belt [19,20]. For a higher level of automation, machine vision is a prospective approach. Machine-vision-driven disassembly utilizes artificial intelligence (AI) techniques and robotics to identify and classify components of interest in the electronic waste and, subsequently, to autonomously disassemble the electronic devices into smaller components. The localization of valuable components via visual information might be of great use for planning and implementing advanced recycling and disassembling strategies.

In addition to commonly used visible light (VIS) and infrared (IR) cameras [21,22,23,24,25], and thermal imaging [26], X-ray radiation has also been used with success in driving the recovery process [27,28,29,30]. By providing real-time feedback on the internal structure, a more optimal disassembly approach can be established, ensuring the efficient separation of the components. X-rays are employed in the recycling of waste electronic equipment for their ability to penetrate deep into the object and provide detailed images of the internal structure of electronic devices in their as-received states. Image segmentation can provide a visual hint about the position of certain components within the item, based on the contrast that arises due to the different local attenuation coefficients. These attenuation coefficients of materials for X-rays are known to depend on the X-ray energy and generally increase from low-Z to high-Z elements and can, therefore, be used for indirect material classification. By training machine learning algorithms with X-ray images, the visual classifier can learn the internal structure and characteristics of different components, and this capability can be utilized for separating various components with different recyclability characteristics, such as metals, plastics, and valuable materials. Considering process safety, X-ray imaging allows for a thorough inspection of electronic components, helping in the assessment of their condition and quality. This is important for identifying components that are suitable for remarketing or those that are broken and may require special handling during the recycling process.

Being a 2D method, X-ray radiography (XR) results in 2D projection images, where both attenuation coefficients and material thicknesses along the raysums determine the contrast in a pixel of the image. A more advanced imaging technique, computed tomography, is capable of separately mapping the 3D volumetric map of the attenuation coefficients. Still, the beam hardening of the radiation as it reaches the deep layers might bias the observed attenuation coefficients, and this makes the material assignation ambiguous for bulky items. Moreover, X-rays might not be selective enough for all material combinations, as the attenuation coefficients of similar materials differ only marginally, and attenuation coefficients of different materials can be coincidently similar. Finally, the tomographic scans need a much longer time compared to radiography, which can be a hindrance in industrial applications. Therefore, due to these constraints, we do not consider tomography further in this study.

Dual-energy X-ray imaging is a possible mitigation for some of these problems [31,32,33]. In this approach, by taking radiograms of the same object using a low-energy and a high-energy voltage setting of the X-ray generator tube, one can already observe different visual contrasts in 2D radiographic projections. For each setting, the negative logarithm of the transmission images results in the product of the linear attenuation coefficients (μ) and the material thickness (*d*) for a pixel at an (*x*,*y*) coordinate (Equation (1)). If the quantity μ differs considerably for the different energy beams, the contrast difference between the two images can be utilized to better classify components, while the thickness dependence can be eliminated according to Equation (2). A combination of imaging techniques based on two different types of particles [34], such as X-ray and neutron imaging [35], can be used in a similar way to dual-energy X-ray imaging.

(1) $$\frac{I}{I_{0}}=e^{-\mu d},\;i.e.,-ln\frac{I}{I_{0}}=\mu d,$$

for X-ray energies *E*_1_ and *E*_2_, or radiation modalities 1 and 2.

(2) $$\frac{{\left[ln\frac{I}{I_{0}}\right]}_{1}}{{\left[ln\frac{I}{I_{0}}\right]}_{2}}=\frac{\mu_{1}}{\mu_{2}}$$

**Figure 1:**
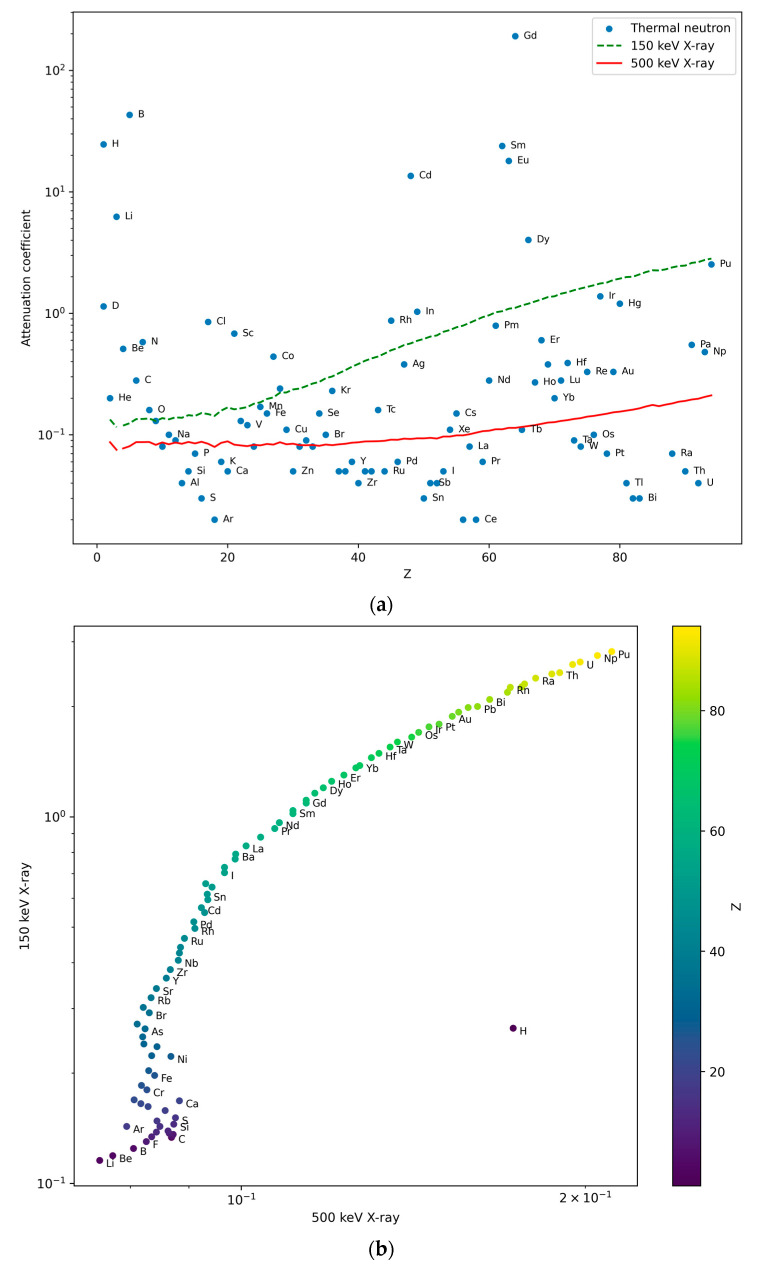

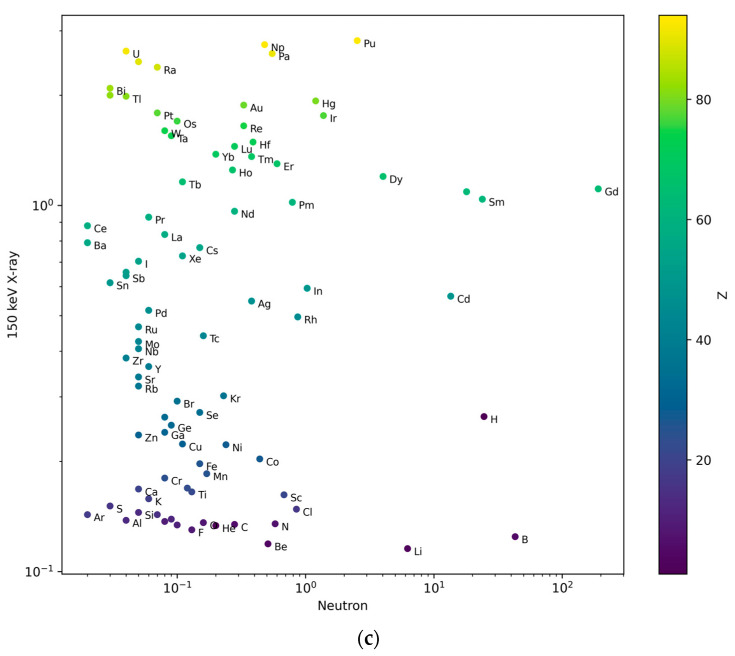
(**a**) Mass attenuation coefficients of thermal neutrons [36] as well as 150 and 500 keV energy X-rays [37] (cm^2^/g) for each element as a function of atomic number. X-ray attenuation curves are smooth but depend on the X-ray energy, while the neutron attenuation largely varies between elements. (**b**) Mass attenuation coefficients for 150 and 500 keV X-rays plotted against each other for each element. (**c**) Mass attenuation coefficient of 150 keV X-rays [37] as a function of the corresponding values for thermal neutrons [36] for each element [37]. Note the different clustering of light elements (lilac), metals (blue), rare earth elements (turquoise blue), and noble metals (grass green) in plots (**b**,**c**), leading to different visual information content about the multi-component items under study.

Neutron imaging, although less common than X-ray imaging, offers unique advantages compared to X-rays, and this may make it appropriate to further develop the above-described approach. Like X-ray imaging, neutron imaging is also non-destructive, allowing for its consideration in the in situ inspection of components. Neutrons can penetrate deep into materials, even if it is challenging for X-rays to pass through, such as heavy metals or high-density materials. This ability allows neutron imaging to provide valuable insights into the internal structure of components that may be difficult to visualize using X-rays alone. Neutrons interact with the atomic nucleus rather than with the electron shell of materials, i.e., the attenuation coefficients vary substantially even between similar materials, leading to enhanced contrast. This can be beneficial for distinguishing between materials with similar X-ray absorption characteristics. Neutron imaging is particularly effective in detecting light elements that are transparent in X-ray imaging, such as hydrogen-containing plastics, or Li-containing battery materials. In addition, it offers high contrast to valuable noble metals (Au, Ag), toxic Cd, and Br compounds, as well as rare earth elements (Eu, Sm, Gd, Dy). This is evidenced in Figure 1a, where the X-ray and thermal neutron mass attenuation coefficients are shown as a function of the atomic number, while in Figure 1b,c, the mass attenuation coefficients of 150 keV X-rays are plotted against the corresponding 500 keV values (these are the typical and the maximum available energies of commercially industrial X-ray generators) and the neutron mass attenuation coefficients, respectively. The simultaneous consideration of the attenuation coefficients for multiple X-ray energies as well as for neutrons offers much higher discrimination power than any of the applied radiation modalities alone.

Neutron-based methods are not only used in non-destructive material testing (NDT) but also in industry [38] on a routine basis. One major application is analyzing material streams for composition [39] in the cement, coal, and mineral industries by an online belt elemental analyzer. During such applications, operation safety and technical aspects of deploying a neutron generator to industrial environments have been successfully addressed [40,41]. X-ray-based imaging techniques have been used repeatedly, according to the literature [27,28,29], for studies aimed at promoting the recycling of various e-waste. On the contrary, the applicability of neutron-based imaging for this purpose is a new aspect that has been scarcely investigated to our knowledge. However, based on its theoretical properties, it has the potential to provide new information about the internal structure that X-ray imaging can only provide to a limited extent or not at all. In this work, we studied the applicability of X-rays and neutrons for the imaging of various WEEE. Our goal was to compare the merits and the limitations of the two standalone imaging techniques, as well as the combination of X-ray and neutron imaging, to achieve a more comprehensive structural characterization of electronic waste items.

## 2. Materials and Methods

### 2.1. Investigated Waste Types

Various types of e-waste such as components of legacy personal computers, including a memory expansion module, a sound card, and a hard drive, as well as a cell phone, a toothbrush, and an electric motor, were investigated. They were studied with neutron and X-ray radiography in their as-received states.

### 2.2. Applied Methods

For neutron radiography (NR), the RAD beamline of the 10 MW thermal power research reactor of the Budapest Neutron Centre (BNC) was used as a neutron source. The beamline is primarily utilized for thermal neutron imaging where the thermal neutron flux is approximately 4.0 × 10^7^ cm^–2^ s^–1^. A significant fast neutron component with a flux around 2.7 × 10^7^ cm^–2^ s^–1^ and a gamma background with about 8.5 Gy/h dose rate were also present. Although our experiments were conducted at the beamline of the Budapest Research Reactor, most of the studies can also be carried out using an industrial neutron generator, applicable even in industrial environments [38,42]. For X-ray radiography, an ERESCO 42 MF3.1 tungsten anode portable industrial X-ray generator device (GE Sensing & Inspection Technologies, presently Waygate Technologies) was applied that could be used in the same bunker and set up with similar imaging geometry. The accelerating voltage and, therefore, the maximum energy of the radiation produced by this equipment could be varied between 25 and 200 keV, with a maximum power of 900 W. The performance of different beam energy settings for the examination of individual WEEE was studied. For each investigated waste, radiography was performed with a preselected optimum beam energy. In each case, the X-ray tube was operated with the maximum current that could be applied to the device at the selected accelerating voltage to operate it at the maximum power, thereby shortening the exposure times. To provide the best reachable spatial resolution (i.e., images as sharp as possible), all the objects were placed as close to the scintillator screen as possible. In this way, we effectively mitigated the geometrical blurring effect of the diverging beams. In such setups, the inherent blur of the scintillator screens is the largest contributor to the blurring effect.

We used a scintillator-based camera system to detect the images. For neutron radiography, a 100 μm thick ^6^LiF/ZnS:Cu scintillation screen was applied to transform neutrons into visible light. In the case of X-ray radiography, this was replaced with a Gadox P43 Gd2O2S:Tb-based OG2 scintillator screen (CAWO solutions, Schrobenhausen, Germany) to convert X-ray photons to visible light. The light pattern of the scintillator was transmitted to the camera by a mirror coated with an aluminum reflective surface, closing an angle of 45° with the beam. A Neo 5.5 (2560 × 2160 pix) sCMOS camera (Andor Technology Ltd. Belfast, Northern Ireland) was used for detecting the image, with a 50 mm or 105 mm focal length objective, depending on the required field of view. The radiograms were stored as 16-bit grayscale TIFF images. The exposure time was set to make use of the full dynamic range of the 16-bit greyscale range but to avoid the saturation of individual pixels. The resulting exposure times were between 3 s and 30 s. The best spatial resolution values reached in both the NR and XR measurements were around 200 ± 50 µm for the 105 mm and the 50 mm focal length setups. As both methods had a similar resolution, from this viewpoint, this does not directly affect the applicability of the two methods compared to each other. At an industrial site, other detector types, such as flat panel detectors, could be even more suitable, but this does not influence the conclusions hereafter.

Open beam images were also taken, which quantified the pixel-wise intensity of the undisturbed neutron or X-ray beam. An additional exposure was performed with the neutron or X-ray beam off, which quantified the dark current of the camera. For each radiogram, at least 3 repeated exposures were made. All recorded radiograms were filtered against outliers using the Fiji-ImageJ software (version 2.15.1) [43], and the median values of the set of three images were computed for each pixel of the final image, to avoid image artifacts and reduce statistical noise. To obtain the final radiograms, the images recorded in the presence of the sample were corrected with open beam images and the dark current of the camera with the image referencing plugin of the Fiji-ImageJ software [43] to eliminate the effects of beam inhomogeneity and electronic noise from the camera.

## 3. Results and Discussion

To assess the advantages and limitations of each imaging method and their combination, the X-ray and neutron radiograms were compared with each other for each type of investigated e-waste. First, printed circuit boards were studied, representing a flat, 2D object geometry.

**Figure 2:**
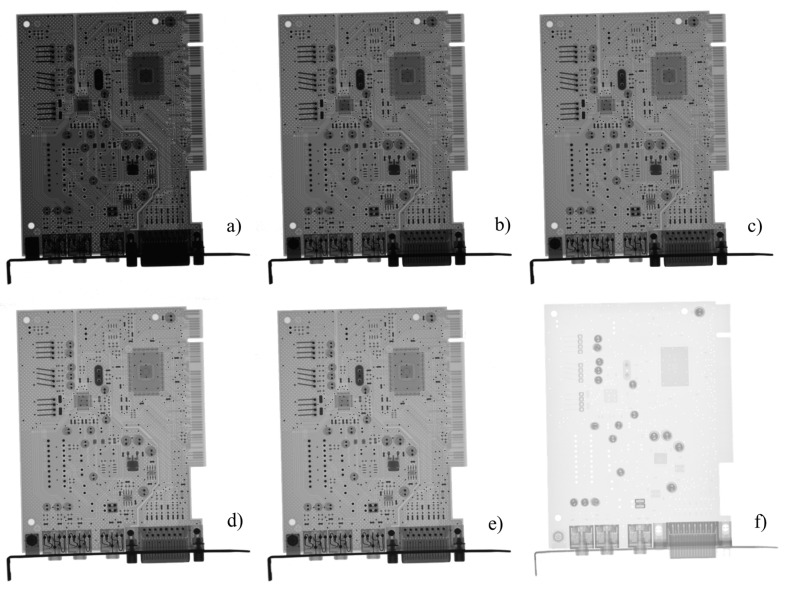
X-ray radiograms of a Creative Sound Blaster PCI 128 type sound card taken with X-ray tube voltages of (**a**) 50, (**b**) 75, (**c**) 100, (**d**) 150, (**e**) 200 kV, and the corresponding (**f**) thermal neutron image. Note that different structural features are visible with the different exposure conditions.

Transmission radiograms (i.e., the 2D projections) of a Creative Sound Blaster PCI 128 (CT4810)-type sound card, taken with multiple X-ray tube accelerator voltages, as well as with thermal neutrons, are shown in Figure 2. In panels (a–e), detailed images of the sound card are presented, where the individual components are clearly distinguishable. The metallic parts were easily differentiated from the non-metallic ones by the human eye. The neutron image, as shown in panel (f), exhibits a substantially different contrast, highlighting the plastic parts and the capacitors. It can be concluded that considering the thickness, material, and structural details of this object, the X-ray images taken at 75 and 100 kV tube voltages were the richest in structural information. At lower energy, the penetration of X-rays was insufficient for a complete screening of the object, while at higher energy beams, the attenuation was not sufficient to have satisfactory contrast to distinguish between the different components of the waste. So, the finer details of the printed circuit board were barely separable from the plastic sheet.

**Figure 3:**
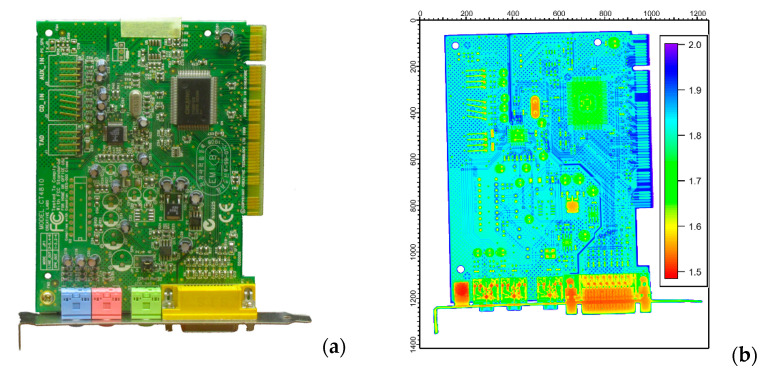
(**a**) The photo and (**b**) the segmentation of the dual-energy X-ray radiogram of the PC sound card. The transmission X-ray radiograms taken at 50 kV and 200 kV were pixel-wise divided and each component was colored according to their typical attenuation values. The axis ticks are given in pixel units.

The most powerful approach for material segmentation is if we correlate information from the different images of the same object and attempt to amplify the differences between pairs (or even sets) of images. The magnitude of variation is representative of a material class. First, dual-energy image pairs with 50 kV and 200 kV X-rays were generated, transformed according to Equation (2), and visualized in Figure 3b. We could observe that the printed circuit board (blue), its copper and gold wiring (light blue), the surface-mounted components (green), and the metallic parts (red) are readily distinguishable.

**Figure 4:**
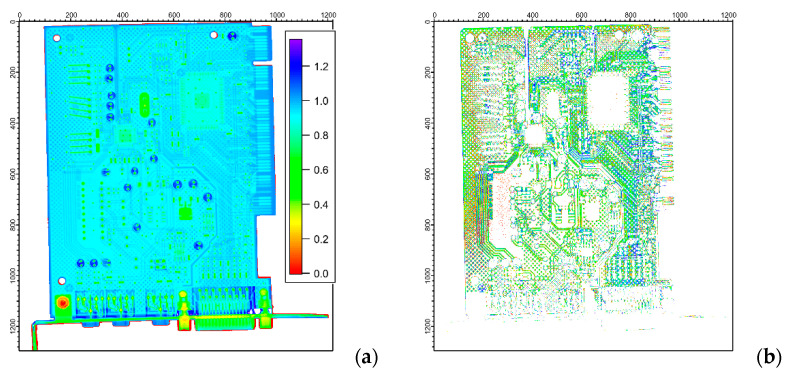

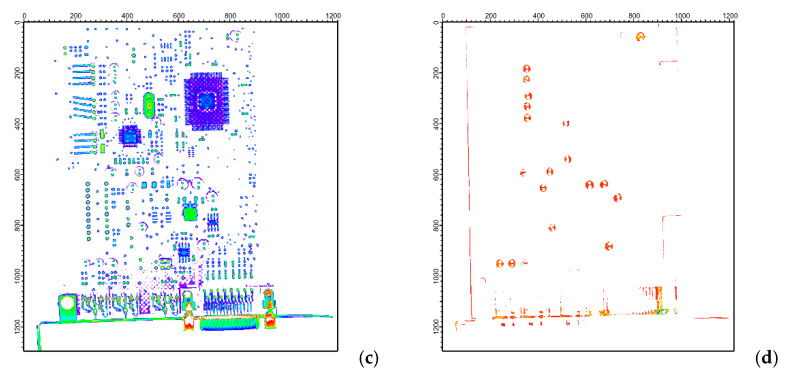
The segmentation of the dual-modality X-ray/neutron radiogram of the PC sound card. Panel (**a**) shows the pixel-wise ratio of the X-ray transmission image to the neutron image without thresholding, while panels (**b**–**d**) show separated structural features of interest, i.e., the wiring of the printed circuit board (**b**), the surface mounted components (**c**) and the capacitors (**d**). The axis ticks are given in pixel units.

An even better segmentation could be achieved when the neutron modality was also considered. This is highlighted in Figure 4, where structural components of interest are also separately plotted using component-specific thresholding. By comparing the dual-energy X-ray image (Figure 3b) and dual-modality neutron/X-ray image (Figure 4a), we can conclude that the neutron/X-ray composite image provides different material separation around the D-Sub socket (see the bottom-right part of the card), while the metal pins, the chips, and the capacitors could also be more quantitatively distinguished.

**Figure 5:**
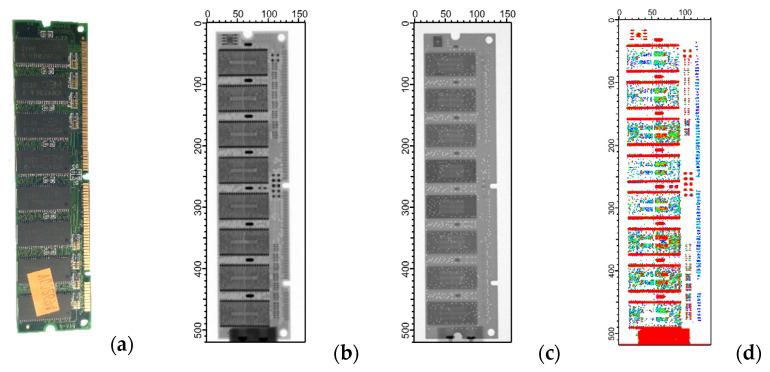
The photo (**a**) and the radiographic images of a PC memory expansion module, taken with (**b**) X-rays at 100 kV accelerating voltage and (**c**) thermal neutrons. The result of the bimodal image processing is on panel, showing the pin contacts in red. The artifact at the bottom of the images is due to an Aluminum support block. (**d**). The axis ticks are given in pixel units.

A similar tendency can be seen on the X-ray and neutron radiograms of a legacy PC memory expansion module, which is shown in Figure 5. Different structural details of the integrated circuits were revealed by the two radiation types. X-ray radiography was also found to be more suitable for visualizing metallic parts, such as welds on the contacts of the integrated circuits. In the neutron image, the ceramic casing of the chips and the glass-fiber PCB substrate are shown prominently. The bimodal image processing was able to selectively locate the pin contacts, shown in red in panel (d).

**Figure 6:**
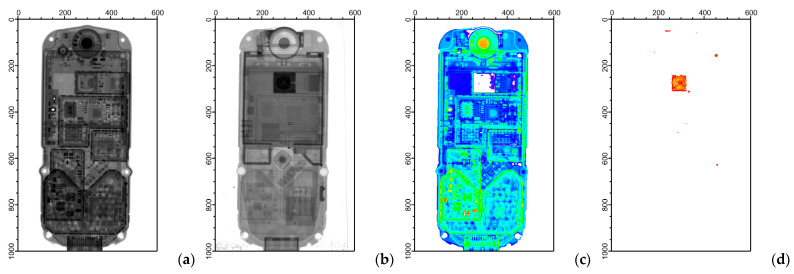
Radiographic images of the mobile phone taken with (**a**) X-rays produced at 200 keV accelerating voltage and (**b**) thermal neutrons. In panels (**c**,**d**), the body (blue), the metallic parts (green), the loudspeaker (orange), and the camera (red) can be separated. The axis ticks are given in pixel units.

Neutron and X-ray radiography images taken from a legacy Siemens mobile phone can be seen in Figure 6a,b. This is a more complex object, containing metals and a plastic body, liquid-crystal display, as well as a PCB part. The two methods were not equally suitable for identifying different components of the phone. In the case of X-ray radiography, metal parts, electronic circuits, capacitors, and aluminum housing were clearly visible with high contrast. It was easy to differentiate between presumably metallic and non-metallic components. On the contrary, the magnet of the loudspeaker, the liquid-crystal display, the camera, and a rubber sealing ring could be prominently seen in the neutron-based image; therefore, neutrons offer a much more efficient possibility for the segmentation of these components. The magnet may contain rare earth elements that have notably high attenuation coefficients for neutrons, while the camera objective might be covered with boron-containing glass, which—unlike X-rays—also attenuates the neutron beam to a high extent. They are both classified as supply-chain-critical constituents.

**Figure 7:**
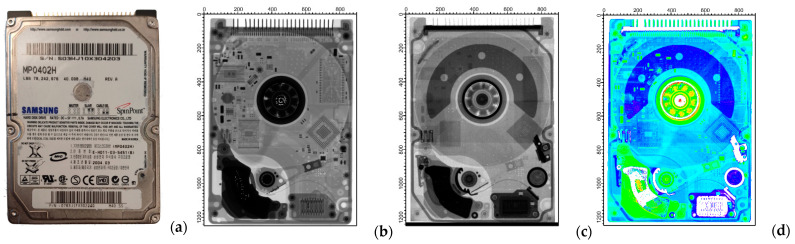
Photo of the hard disk (**a**). Radiograms of the hard disk taken with (**b**) X-rays at 100 kV voltage and (**c**) thermal neutrons. The composite image (**d**) exhibits information from both modalities. Note that the white areas are inconclusive regions due to complete absorption of one or other radiation type. The axis ticks are given in pixel units.

Figure 7 shows a photograph (a), the X-ray (b), and the neutron radiograms (c) of a 2.5″ Samsung laptop hard disk, representing a further step in complexity. Similarly to the mobile phone, both methods were found to be capable of identifying some of the inner structure of this waste and contributing to the material segmentation. However, some components were only visible on the neutron radiograms, while other constituents were visualized on the X-ray radiography images. For example, various presumably metallic parts and circuit components were much better contrasted in the X-ray radiograms. Some other constituents, e.g., the arch-shaped item at the top of the image and the circular-shaped component at the lower-right side of the image, showed little contrast or no contrast at all on X-ray radiography images but were highly contrasted on neutron radiograms. Similar to the mobile phone, the combined interpretation of the two methods indicates that materials with high neutron attenuation were responsible for this. So, as before, it could also even indicate the presence of different elements important for the circular economy, such as various rare earth elements.

We observed some parts that gave good contrast with both neutron and X-rays. According to the literature, the most typically used magnet in hard disks is the neodymium magnet. This is an alloy with a stoichiometric composition of Nd_2_Fe_14_B [44]. In some cases, a minor part of the neodymium is replaced by dysprosium to improve the properties of the magnet [45]. Both the main component, neodymium, and the possible minor component, dysprosium, are important critical raw materials. Both rare earth elements have high X-ray and neutron attenuation coefficients. The main component of the magnet is iron, which has medium attenuation capability for both radiation types. The third component of this type of magnet is boron, which has a remarkably high neutron absorption coefficient, while its X-ray attenuation is almost negligible. These theoretical considerations suggest a remarkably high attenuation against both neutron and X-ray radiation in the region of the magnetic components. Consequently, parts that are well attenuated on both types of radiograms, based on these considerations, can indicate the presence of neodymium and dysprosium. The presence of these aforementioned elements was subsequently confirmed by prompt-gamma activation imaging experiments. The detailed results of these studies will be published separately.

**Figure 8:**
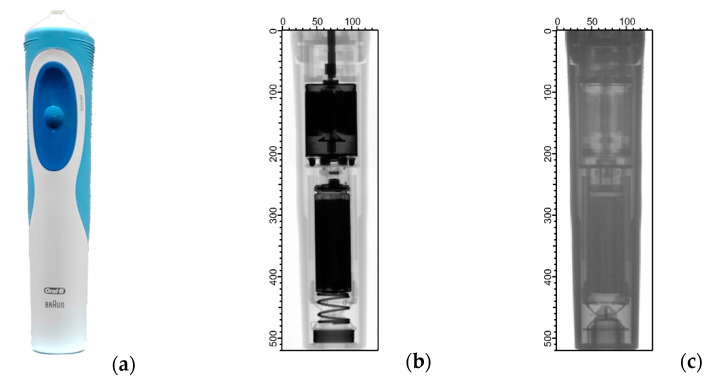
The photo (**a**) and the radiographic images of the electric toothbrush, taken with (**b**) X-rays produced at 150 kV tube voltage and (**c**) thermal neutrons. The axis ticks are given in pixel units.

Unlike the so-far-presented computer parts, the next group of investigated items is cylindrical geometries. The neutron and X-ray radiograms of a Braun Oral-B electric toothbrush are visualized in Figure 8. It can be concluded that the images obtained by thermal neutron radiography were found to have lower contrast and poorer spatial resolution than by X-rays. The reason for this is the high hydrogen content of the plastic used in the external cover of the toothbrush, as well as present in the battery. The hydrogen in the plastic and the battery is an element that has a large neutron scattering cross-section so that its presence in large amounts can result in low neutron transmission, as well as deteriorated spatial resolution in neutron radiography images if placed close to the image detector. The obtained X-ray radiograms were of much better contrast this time, and the internal structure of the electric toothbrush, the battery, and the electric motor were visible. The information in the two images is complementary and helps to separate the organic and metallic constituents of the appliance. It can, therefore, also be used to identify a safety risk related to leaking batteries.

**Figure 9:**
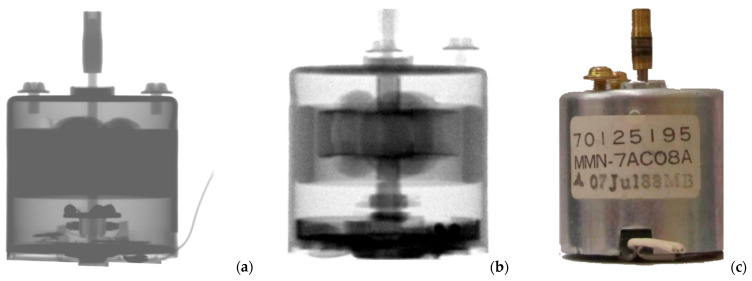
Radiograms of an electric motor taken with (**a**) X-rays produced at 100 kV X-ray voltage and (**b**) thermal neutrons. (**c**) Photo of the electric motor.

On the other hand, although the toothbrush contains an electric motor of about 15 mm in diameter, this is already challenging to transilluminate with moderate-energy X-rays (see between 100 and 180 px in Figure 8a). This results in a local loss of information. It becomes an increasing problem as the projected thickness of the object under study becomes larger than the penetration depth of the applied radiation. To confirm this, the radiograms and a photo of an electric motor of about 30 mm are presented in Figure 9. Here, neutron radiography (Figure 9b) could still successfully reveal the internal structure of the iron core and the copper wiring part of the rotor that falsely appeared homogeneous by X-ray imaging (Figure 9a). This demonstrates the higher penetration power and the different material discrimination ability of neutrons compared to X-rays, which is advantageous if items with thick metallic components are studied. For another part of the waste, though, the X-ray radiography provided more detailed information (see the bottom half of the images).

## 4. Conclusions

We successfully demonstrated that both X-ray and neutron radiography is a suitable technique for the determination of the internal structure of different e-waste types and the identification of their constituents. Because of the deep penetration power of neutrons and X-rays, these radiography techniques generally provide a wider range of information than many other surface-confined techniques used in the literature, such as visible light (VIS) or infrared (IR) cameras, and thermal imaging. This volume-representative information can be a useful input for future, even industrially applicable, intelligent waste sorting and disassembly systems.

For both methods, we identified some limitations for the determination of the structure of the investigated e-waste. However, the two methods often provided complementary information on the structure. One of the main limitations of X-rays is the low maximum penetration depth to certain materials; thus, the transparency of thicker metallic parts was strongly limited. Some other constituents, on the contrary, gave little or no contrast with X-ray radiography. These parts were typically much more visible with neutron-based imaging. With neutron radiography, however, the identifiability of some other parts, primarily small metallic parts, was limited, e.g., the finer details of a few microns thick printed electronic circuits were poorly or not visible; these components could appear in much more detail and with better contrast on the X-ray radiograms. Another limitation of neutron-based imaging was observed in the case of plastic-containing WEEE, where large amounts of hydrogen may create excessive beam attenuation and scattering, resulting in poorly contrasted and blurred images, while in X-ray images, the plastics show up weakly due to the negligible attenuation of X-rays in the polymer matrix composed of light elements so that the structure under the polymer coating was more visible.

We proved that more comprehensive information could be obtained about the internal structure of the different WEEE by applying the combination of the neutron and X-ray imaging techniques than by either radiation modality alone. The combination of the methods was especially advantageous for e-waste with highly complex structures such as mobile phones and hard disks. Each method is “blind” to certain materials while being able to clearly visualize others, meaning a good complementarity, which finally leads to a more effective material segmentation. We also demonstrated that dual-energy X-ray imaging could achieve more efficient segmentation than radiography based on a single X-ray energy, but neutron radiography was also an effective complement to this technique, providing segmentation possibilities that could not have been achieved with dual-energy X-ray imaging alone.

By interpreting the two radiographic methods together, it was possible to identify components that had a significantly different composition from their surroundings. When regions exhibit a certain contrast enhancement factor, they can indicate constituents relevant to the circular economy at elevated concentrations. For instance, it may indicate the local enrichment of rare earth elements**,** considered as critical elements.

The result obtained by combining and interpreting the two methods together has the potential to serve as an input for an automated disassembly process on a production line, which can disassemble and selectively collect parts of e-waste with significantly different elemental compositions. By collecting these parts separately from the rest of the waste items, the environmental footprint of the subsequent processing and recovery steps could be reduced due to the significantly higher concentration of the elements to be recovered in the separated components compared to the original e-waste. Our long-term vision is that a combination of the two techniques could be implemented directly at the place of disassembly using industrial neutron and X-ray generators. However, the assessment of the transferability of the methods to an industrial environment is beyond the scope of this study. In the future, we intend to investigate the possibilities for industrial applications. However, the combination of the two imaging methods is only suitable for the identification of parts with rather different elemental compositions. It is not applicable for the direct and quantitative determination of the elemental composition, for which, if required, there exist methods, such as portable X-ray fluorescence spectroscopy, that can even be used in situ in complement to imaging techniques. If such a pXRF device is mounted on a robotic arm, it can be positioned based on the acquired images to provide quantitative composition assessment of the components of interest. As a continuation of this research, we also foresee further studies on the possibilities of combining imaging methods with in situ elemental composition analytical techniques.

## Data Availability

The original contributions presented in the study are included in the article, further inquiries can be directed to the corresponding authors.
